# The mutational landscape of spinal chordomas and their sensitive detection using circulating tumor DNA

**DOI:** 10.1093/noajnl/vdaa173

**Published:** 2020-12-08

**Authors:** Austin K Mattox, Beibei Yang, Christopher Douville, Sheng-fu Lo, Daniel Sciubba, Jean Paul Wolinsky, Ziya L Gokaslan, Jamie Robison, Cherie Blair, Yuchen Jiao, Chetan Bettegowda

**Affiliations:** 1 Ludwig Center for Cancer Genetics and Therapeutics, Sidney Kimmel Comprehensive Cancer Center, Johns Hopkins University School of Medicine, Baltimore, Maryland, USA; 2 State Key Lab of Molecular Oncology, National Cancer Center/National Clinical Research Center for Cancer/Cancer Hospital, Chinese Academy of Medical Sciences and Peking Union Medical College, Beijing, China; 3 Department of Neurosurgery, Johns Hopkins University School of Medicine, Baltimore, Maryland, USA; 4 Department of Neurosurgery, Northwestern University School of Medicine, Chicago, Illinois, USA; 5 Department of Neurosurgery, Brown University School of Medicine, Providence, Rhode Island, USA; 6 Department of Neurosurgery, Mayo Clinic, Jacksonville, Florida, USA; 7 Department of Oncology, Johns Hopkins University School of Medicine, Baltimore, Maryland, USA

**Keywords:** biomarker, cell free DNA, chordoma, circulating tumor DNA, liquid biopsy

## Abstract

**Background:**

Chordomas are the most common primary spinal column malignancy in the United States. The aim of this study was to determine whether chordomas may be detected by evaluating mutations in circulating tumor DNA (ctDNA).

**Methods:**

Thirty-two patients with a biopsy-confirmed diagnosis of chordoma had blood drawn pre-operatively and/or at follow-up appointments. Mutations in the primary tumor were identified by whole exome sequencing and liquid biopsy by ddPCR and/or RACE-Seq was used to detect one or more of these mutations in plasma ctDNA at concurrent or later time points.

**Results:**

At the time of initial blood draw, 87.1% of patients were ctDNA positive (*P <*.001). Follow-up blood draws in twenty of the patients suggest that ctDNA levels may reflect the clinical status of the disease. Patients with positive ctDNA levels were more likely to have greater mutant allele frequencies in their primary tumors (*P* = .004) and undergo radiotherapy (*P* = .02), and the presence of ctDNA may correlate with response to systemic chemotherapy and/or disease recurrence.

**Conclusions:**

Detection of ctDNA mutations may allow for the detection and monitoring of disease progression for chordomas.

Key Points

- Chordomas may be detected by evaluating mutations in plasma ctDNA.
- ctDNA levels may correlate with response to adjuvant therapy and/or recurrence.

**Importance of the Study**

Chordomas are the most common primary spinal column malignancy. Extensive surgical procedures, post-operative radiation, and neoadjuvant chemotherapy are often required to reduce the chances of recurrence. Currently, CT, MRI, and PET scanning are used to monitor for disease recurrence but can be limited by surgical sequalae. In addition, needle biopsy runs the risk of tumor seeding along the biopsy track. We describe the mutational landscape of spinal chordomas and show that ctDNA is a sensitive biomarker for detecting disease and has potential for integration into clinical care.

Chordomas are the most common primary spinal column malignancy with an incidence of one to two cases per million in the United States.^1^ They are gelatinous, grayish, and typically encapsulated tumors that originate from extradural remnants of the notochord.^2^ They typically occur in the 40- to 60-year old age range and have a higher prevalence in men than in women.^1^ The treatment for spinal chordomas often involves extensive surgical procedures requiring *en bloc* resection with negative margins. In some cases, neoadjuvant or post-operative radiation is utilized to minimize chances of recurrence. Despite aggressive multimodal treatment regimens, median progression free survival is only five years and overall survival is seven years.^3^ Local recurrence remains a major challenge. More than 30–50% of patients develop recurrence locally, and about 10–20% go on to develop disseminated disease, often in the lungs or liver.^4^ One of the principle challenges in caring for individuals with chordoma is identifying those patients that are at highest risk for recurrence, as post-operative radiotherapy and/or chemotherapy is most appropriate for these individuals.

Recently, an international collaborative group demonstrated that patients lacking the adenine variant of the germline SNP rs2305089 in the brachyury gene are at higher risk for poor outcome, identifying a subset of patients likely to recur. The same group also found that 9% of spinal chordomas harbor canonical C228T and C250T mutations in the hTERT promoter region. The patients whose tumors contain the mutations had significantly improved survival over those without the mutation.^5^ Unfortunately, only a small subset of patients will lack the A allele at SNP rs2305089 or harbor the hTERT promoter mutation, suggesting that there are additional factors playing a role in recurrence and metastasis.^3^ The current means for monitoring individuals with known chordoma involves conventional imaging modalities including CT, MRI, and/or PET scanning.^2^ Given the extensive surgical procedures often performed for chordomas, radiographic findings can be limited by hardware used to stabilize the spine, anatomic changes secondary to complex tissue reconstructions, and treatment changes associated with radiation or chemotherapy. In addition, performing percutaneous needle biopsies of suspicious lesions can be invasive and risks tumor seeding along the biopsy track.

As a result, there exists an urgent need to develop minimally invasive biomarkers to help track disease burden in individuals with chordoma (Figure 1). Our group and others have shown that many human tumors will shed molecules of tumor DNA in the bloodstream (ctDNA).^6–8^ ctDNA harbors tumor specific somatic mutations that can be used to differentiate it from the abundant DNA derived from normal cells. Levels of ctDNA can be quantified and tracked over time for disease recurrence and therapeutic monitoring. Recent data suggest that ctDNA can detect microscopic disease burden 6 months prior to conventional imaging in certain cancers.^6,7^ To date, there is no literature that has tested whether chordomas shed tumor derived DNA into the circulation. To help address this we performed a proof-of-principle prospective study in individuals undergoing surgical resection of a spinal chordoma at a single institution to determine whether mutations in ctDNA were detectable in the plasma. Blood was collected and processed pre-operatively from 32 patients and in subsequent follow-up time points in 20 patients. Surgeons and treating physicians were blinded to the results of the study, and study outcomes did not affect clinical course or treatment. In order to identify a somatic mutation within the tumor, matched chordoma and normal tissue was subjected to whole exome sequencing. Once a somatic alteration was identified in the tumor, specific droplet digital PCR (ddPCR) primers and/or rapid amplification of cDNA ends sequencing (RACE-Seq) PCR primers were developed and ctDNA mutation levels were assessed in the plasma.^9,10^ The levels of tumor DNA were correlated with radiographic and clinical findings.

**Figure 1.**
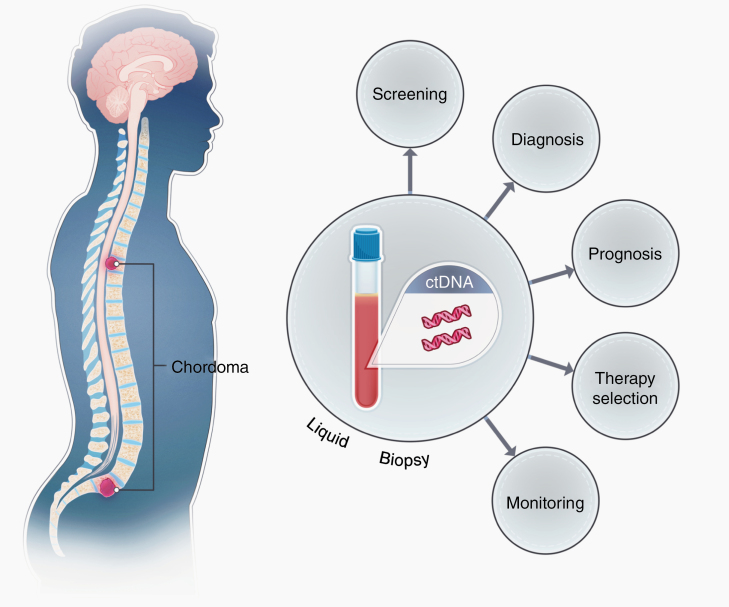
Schematic demonstrating the shedding of ctDNA derived from spinal chordomas in the plasma of patients. Chordomas shed DNA containing mutations into systemic circulation, allowing for non-invasive screening and recurrence monitoring by liquid biopsy techniques such as ddPCR and RACE-Seq. Prognostic and/or therapeutic information may also be imparted by liquid biopsy, depending on the nature of the mutation identified.

## Materials and Methods

### Patients

Thirty-two patients undergoing surgical resection of a spinal chordoma at the Johns Hopkins Hospital (JHH) provided informed consent to participate in an IRB approved protocol (IRB00075499). All patients with biopsy-confirmed diagnosis of a primary spinal chordoma were eligible for the study. Clinical and radiographic measures of disease were documented at the initial and subsequent visits with clinicians at JHH. Twenty patients returned for follow-up and contributed post-operative blood samples. Twelve patients did not return to JHH or declined further participation in the study. Clinicopathological features of the patient cohort are found in Table 1 and Supplementary Table 1.

**Table 1.** Clinicopathological Characteristics of the Patient Cohort

Characteristics	All Patients	ctDNA Positive	ctDNA Negative	*P* Value
Median age at diagnosis (range)	53.3 (25.1–74.7)	53.0 (25.1–67.7)	58.3 (34.6–74.7)	.48
Sex, no. (%)				
Male	20 (62.5)	16 (59.3)	3 (75.0)	.55
Female	12 (37.5)	11 (40.7)	1 (25.0)	.55
Primary tumor site, no. (%)				
Chest (metastatic from spine)	1 (3.1)	1 (3.7)	0 (0.0)	.70
Pelvis	2 (6.3)	1 (3.7)	1 (25.0)	.11
Sacrum	15 (46.9)	13 (48.1)	2 (50.0)	.94
Spine	14 (43.8)	12 (44.4)	1 (25.0)	.46
Treatment modality, no. (%)				
Surgery	32 (100.0)	27 (100.0)	4 (100.0)	.99
Radiotherapy	8 (25.8)	5 (18.5)	3 (75.0)	.02
Chemotherapy	1 (3.2)	1 (3.7)	0 (0.0)	.70
Radiographic features				
Tumor burden at diagnosis in mm^3^, median (range)	39.9 (1.1–5712)	36.0 (1.1–5712)	101.7 (36.4–178.8)	.58
Local recurrence at initial blood draw, no. (%)	4 (12.5)	3 (11.1)	1 (25.0)	.44
Metastatic disease at initial blood draw, no. (%)	1 (3.1)	1 (3.7)	0 (0.0)	.70
Genomic features				
Number of unique exome mutations, median (range)	5.5 (1.0–74.0)	5 (1.0–74.0)	23 (14.0–27.0)	.47
Whole exome sequencing mutant allele fraction (%), median (range)	25 (4.1–87.5)	28 (5.0–87.5)	21.2 (7.6–56.0)	.004
ctDNA mutant allele fraction (%), median (range)	0.2 (0.0–29.5)	0.2 (0.0–29.5)	0.0 (0.0–0.0)	.35
Positive liquid biopsy at initial blood draw, no. (%)	27 (87.1)	–	–	.001
Length of follow-up in months, median (range)	24.2 (0.0–107.9)	24.2 (0.0–107.9)	31.7 (14.1–93.5)	.62

### Sample Preparation

***Library construction and whole exome sequencing***Tumor and matched lymphocytic normal DNA library preparation was performed as previously described.^11^ Genomic DNA from tumor and normal samples were fragmented and used for Illumina TruSeq library construction (Illumina, San Diego, CA) according to the manufacturer's instructions. Detailed buffer, PCR, and interval purification conditions are described in the Supplementary Methods.

### Plasma Preparation

Plasma was isolated from whole blood as previously described.^6^

### Assay Methods

***Processing of next-generation sequencing data***Somatic mutations were identified using VariantDx custom software for identifying mutations in matched tumor and normal samples from whole exome sequencing (WES). Prior to mutation calling, primary processing of sequence data for both tumor and normal samples were performed using Illumina CASAVA software (v1.8), including masking of adapter sequences. Sequence reads were aligned against the human reference genome (version hg18) using ELAND software. Candidate somatic mutations, consisting of point mutations, insertions, and deletions were then identified using VariantDx. In brief, an alignment filter was applied to exclude quality failed reads, unpaired reads, and poorly mapped reads in the tumor. A base quality filter was applied to limit inclusion of bases with reported phred quality score >30 for the tumor and >20 for the normal. A mutation in the tumor was identified as a candidate somatic mutation only when (i) distinct paired reads contained the mutation in the tumor; (ii) the number of distinct paired reads containing a particular mutation in the tumor was at least 10% of the total distinct read pairs; (iii) the mismatched base was not present in >1% of the reads in the matched normal sample as well as not present in a custom database of common germline variants derived from dbSNP; and (iv) the position was covered in both the tumor and normal. Mutations arising from misplaced genome alignments, including paralogous sequences, were identified and excluded by searching the reference genome.

Candidate somatic mutations were further filtered based on gene annotation to identify those occurring in protein coding regions. Functional consequences were predicted using snpEff and a custom database of CCDS, RefSeq, and Ensembl annotations using the latest transcript versions available on hg18 from UCSC (https://genome.ucsc.edu/). Predictions were ordered to prefer transcripts with canonical start and stop codons and CCDS or Refseq transcripts over Ensembl when available. Finally, mutations were filtered to exclude intronic and silent changes, while retaining mutations resulting in missense mutations, nonsense mutations, frameshifts, or splice site alterations. A manual visual inspection step was used to further remove artifactual changes.

***ddPCR***Cell-free DNA was extracted using the QIAGEN circulating nucleic acid kit (Catalog# 55114). Extracted cell-free DNA was analyzed with custom designed droplet digital PrimePCR™ assays using the BioRad QX200 droplet digital PCR system to determine the number of wild-type and mutant genomic equivalents following the manufacturer's recommendations. The sequences of the primers are found in Supplementary Table 2. A mutation was selected for each tumor based on the results of the whole exome sequencing (WES) results. ddPCR was then performed on DNA derived from the plasma and ctDNA levels were quantified. These data were used to calculate the overall mutant allele frequency (MAF) for each somatic mutation, defined as the number of mutant counts divided by the total number of counts for a given amplicon. All ddPCR assays were conducted blinded to all study endpoints described in Table 1. If only one mutation was identified by ddPCR in plasma, the sample was called positive if the mutation had three or more counts. If two or more mutations were identified by ddPCR in plasma, the sample was called positive if there were two or more mutant counts in at least one of the amplicons, in addition to one or more mutant counts in at least one or more amplicons.

***RACE-Seq***The somatic mutations identified in tumor tissue by exome sequencing were tracked by RACE-Seq to determine its fraction in the matched ctDNA sample as previously described.^12^ Briefly, ctDNA was ligated to an adaptor with random DNA barcodes with KAPA Hyper Prep Kit (KK8500 KAPA Biosystems, Roche, MA). The ligated constructs were amplified for a whole genome library. The target regions harboring the somatic mutations in tumor were enriched with a universal primer matching the adapter sequence and a target-specific primer (TS primer 1), as listed in Supplementary Table 2. A second round PCR with one pair of nested primers matching the adapter and the target region (TS primer 2, Supplementary Table 2) was used to further enrich the target region and add the full length of Illumina adapter. The libraries were sequenced with an Illumina NovoSeq 6000 Sequencing System. The DNA barcode allows tracking of redundant reads from an original ctDNA molecule to minimize false positive mutations from PCR amplification and sequencing error. The fraction of a mutation in ctDNA was determined by the mutant read clusters (original ctDNA molecules) and the wild type read clusters.

***RealSeqS***In cases where there was no bona fide somatic mutation identified in the tumor, we used RealSeqS to test the plasma for levels of aneuploidy as a measure of ctDNA. RealSeqS uses a single primer pair to amplify about 750,000 loci scattered throughout the genome.^13^ After massively parallel sequencing, gains or losses of each of the 39 chromosome arms covered by the assay were determined using a bespoke statistical learning method.^14^ A support vector machine (SVM) was used to discriminate between aneuploid and euploid samples. The SVM was trained using 2651 aneuploid samples and 1348 euploid plasma samples. Samples were scored as positive when the genome-wide aneuploidy score was >0.441.

Plasma samples were also analyzed for genomic DNA contamination using RealSeqS. RealSeqS enables the detection of genomic DNA by virtue of the differently-sized amplicons generated during PCR amplification. Reads at the 1241 amplicons described in Douville *et al.* indicates the presence of genomic DNA.^13^

### Study Design

This was a prospective study designed to detect mutations in the ctDNA of patients with a diagnosis of biopsy-confirmed spinal chordoma seen at JHH from August 2012 to August 2018. As chordomas are rare CNS tumors with a clinical course that manifests over several years, we aimed to recruit all patients eligible for the study over the six-year period. Of the 79 patients seen for a diagnosis of spinal chordoma at Johns Hopkins, we successfully recruited 32 (41%). As this is the first study designed to detect ctDNA mutations in the plasma of patients with a history of biopsy-confirmed chordomas, we did not have *a priori* knowledge to power the study to detect a specific effect size. Patients were not stratified by disease stage, age, or other clinicopathological characteristic. Median follow-up time was 24.2 months by the end of the follow-up period was December 12, 2019. The primary endpoint was detection of mutations in ctDNA. Clinical variables collected included site of primary tumor, date of surgery, gender, age, tumor volume at time of surgery, presence or absence of radiographically-identified locally recurrent disease at the time of each blood draw, presence or absence of radiographically-identified metastatic disease at the time of each blood draw, date of last follow-up, presence or absence of radiographically-identified disease at the time of last follow-up, adjuvant radiotherapy and/or chemotherapy, and positive or negative liquid biopsy test.

### Statistical Analysis Methods

Clinicopathological characteristic comparison statistics between ctDNA positive and ctDNA negative samples were conducted by two-tailed Student's *t*-test for age, follow-up time, tumor burden at initial surgery, number of unique exome mutations, WES mutant allele fraction, and ctDNA mutant allele fraction. The Chi Squared test was used for sex, tumor location, radiotherapy, systemic therapy, presence of locally recurrent disease at initial blood draw, presence of metastatic disease at initial blood draw, and liquid biopsy result at initial blood draw. A *P*-value less than or equal to .05 was considered significant. A list of mutations and their frequencies was submitted to DBString for functional annotation clustering.^15^ Pathways were considered significant when their false discovery rate (FDR) was less than 0.01.

## Results

### Data

Thirty-two adult patients undergoing surgical resection of spinal chordomas were enrolled in this study, including 27 patients (84.4%) who presented at the time of initial diagnosis, 4 patients (12.5%) who presented with locally recurrent disease, and 1 patient (3.1%) who presented with recurrence at the primary site and a metastatic lesion to the chest. All 32 patients underwent gross total resection of their tumors with negative margins based on pathological examination. Clinicopathological characteristics of the patient cohort are listed in Table 1.

**Figure 2.**
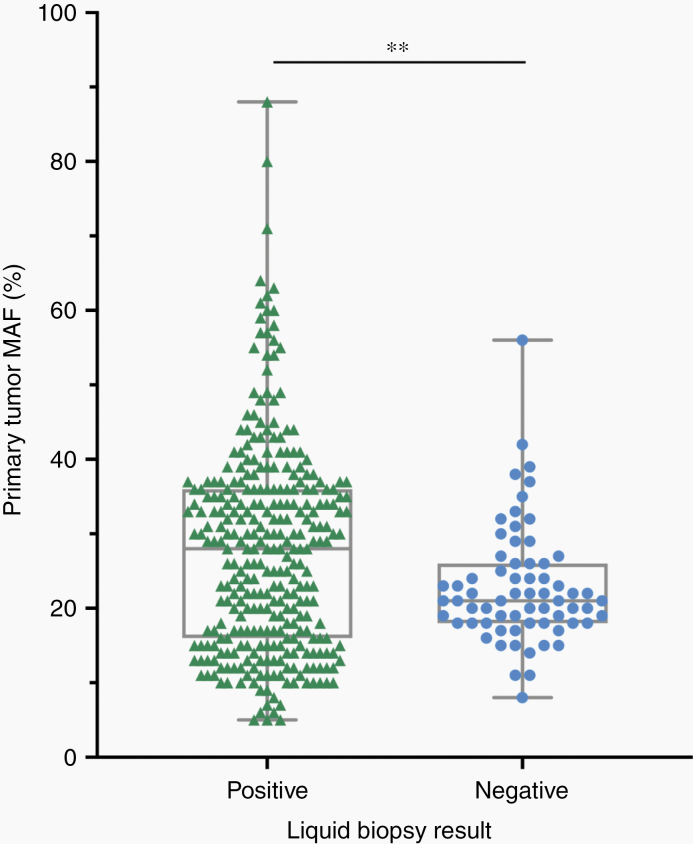
Plasma samples that were positive for concordant mutations in ctDNA were more likely to have greater MAFs identified in the primary tumor by whole exome sequencing. Compared to liquid biopsy negative plasma samples, plasma samples that scored positive for one or more ctDNA mutations of interest had greater MAFs identified in the primary tumor. The data points are presented as the median, minimum, and maximum. Student's two-tailed *t*-test for differences between MAFs in the primary tumor: **P <.01.

For 30 of the 32 patients, blood was drawn before surgical resection and collected at routine follow-up appointments where possible. Blood draws for two of the patients were not available prior to surgery and were collected at follow-up appointments after surgical resection. Patients who did not provide subsequent blood samples often chose to attend follow-up appointments at outside hospitals in their local area. Twelve of the 32 patients were women (37.5%), and the patients had a median age of 53.3 years. Tumors were most likely to be diagnosed in the sacrum (46.9%) or spine (43.8%) but were also found in the pelvis (6.3%) or metastatic to the chest from a spinal primary (3.1%). Median follow-up time was 24.2 months, and 25.8% of patients received radiotherapy and 3.2% of patients received systemic chemotherapy. Median tumor burden at the time of surgery, measured upon pathological examination, was 39.9 mm^3^. Nearly all clinicopathological characteristics were similar between patients that were ctDNA positive vs ctDNA negative at initial blood draw, except ctDNA positive patients were more likely to have higher mutant allele fractions detected in their primary tumors (*P* = .004) and to receive radiotherapy (*P =* .02) than ctDNA negative patients (Figure 2).

### Analysis and Presentation

*The mutational landscape of primary spinal chordomas*Whole exome sequencing (WES) was performed on the primary tumor for each of the 32 patients enrolled in the study. Across all patients, we identified 379 somatic mutations with a median of 5.5 mutations per patient (Table 1). The median mutant allele frequency (MAF) for an identified mutation was 25%, and patients that were positive by ctDNA analysis were more likely to have a higher MAF than those that were ctDNA negative (*P* = .004, Two-Tailed Student's *t-*Test). The vast majority of mutations were single base substitutions (92%), but a few tumors contained detectable indels. Most mutations resulted in nonsynonymous amino acids changes (79.5%) or the creation of frameshift (7.8%) or nonsense (6.8%) mutations (Supplementary Figure 1). Mutation type distribution did not differ between ctDNA positive and ctDNA negative tumors.

**Figure 3.**
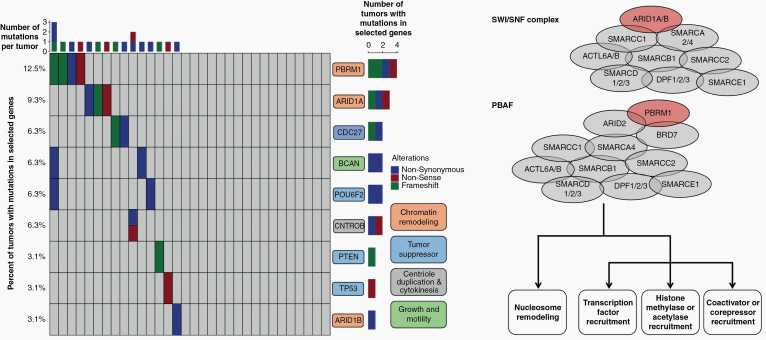
The primary tumors of spinal chordomas demonstrate recurrent mutations in tumor suppressors, and genes involved in chromatin remodeling, centriole duplication and cytokinesis, and growth and motility. Twenty-five percent of tumors tested had a mutation in genes that regulate epigenetic status across the genome, including mutations in genes responsible for chromatin remodeling and histone methylation. Most notable were mutations in *ARID1A, ARID1B,* and *PBRM1* (colored red in the schematics for the SWI/SNF complex and PBAF complex), key members of the SWI/SNF and PBAF complexes that play a role in chromatin structure and transcriptional regulation. Loss of SWI/SNF or PBAF complex members may lead to transcriptional dysfunction, including disruption of nucleosome sliding activity, affecting the ability of the RNA polymerase II complex to bind promoters across the genome.

Interestingly, there were recurrent somatic mutations in genes that encode epigenetic machinery, including *ARID1A, ARID1B,* and *PBRM1* in 25% of tumors (Figure 3, Supplementary Table 3). Other recurrent mutations included genes encoding BCAN, a protein highly expressed in gliomas that may promote the growth and cellular motility of tumor cells, CNTROB, a protein required for proper centriole duplication and cellular cytokinesis whose mutation can lead to aneuploidy, and mutations in the tumor suppressor genes *PTEN, TP53, POU6F2,* and *CDC27* (a component of the APC complex). Functional annotation of the protein networks impacted by these mutations highlighted pathways involved in chromatin remodeling (FDR = 0.0016) and histone methylation (FDR = 0.0089).

A recent study by Tarpey *et al*. conducted either whole genome or whole exome sequencing on 26 spinal chordomas^16^. Our analysis of 32 spinal chordomas identified alterations in *LYST*, *PTEN*, *CDC27*, and *ARID1A* at similar frequencies compared with their spinal chordoma cohort. While robust statistical analysis is limited by sample size given the rarity of the tumors, we identified more mutations in *PBRM1* compared with the discovery cohort of Tarpey *et al.* (*P = .06*). Additionally, to characterize the molecular landscape of spinal chordomas compared to chordomas arising from other sites, we also compared our findings against the whole genome or exome data of non-spinal chordomas sequenced in the discovery cohort of the work by Tarpey *et al.* We identified no significant differences in the mutational spectra of spinal versus non-spinal chordomas, suggesting they are similar to chordomas in other locations.

*Sensitive detection of primary spinal chordomas in plasma*ddPCR or RACE-Seq was performed on the matched plasma based on the WES results for at least one mutation per tumor specified in Supplementary Table 3. The initial set of 15 samples was amplified using ddPCR primer/probe sets that were developed to amplify the genomic locus of the mutation identified as the greatest MAF in the primary tumor by whole exome sequencing. At the time of amplifying the next set of 17 samples, we chose to use a newer technology, RACE-Seq, in order to assay for multiple mutations simultaneously at the initial blood draw and follow-up timepoints. The mutations assayed at each blood draw, the identified plasma MAF, and whether the liquid biopsy was considered positive or negative can be found in Supplementary Table 4. From the 32 confirmed chordoma cases, at least one amplicon was successfully amplified from plasma ctDNA. Of the 31 cases that could be interpreted, 27 had positive ctDNA results based on one or more identified mutations, giving an overall sensitivity of 87.1% at the time of initial blood draw (Table 1). The median mutant allele fraction in cases with detectable levels of ctDNA was 0.2%.

Twenty of the thirty-two patients had at least one follow-up blood draw performed after surgery (Supplementary Tables 1 and 4). If ddPCR was used at the time of the initial blood draw to detect the candidate mutation from WES in the plasma, we also used ddPCR to assay for this same mutation at later time points. Unlike ddPCR, RACE-Seq permits simultaneous analysis of multiple mutations. Therefore, when RACE-Seq was employed, we attempted to assay for all somatic mutations detected in the primary tumor via WES. On average we queried for three to four mutations in the plasma at the time of initial blood draw and all subsequent time points using RACE-Seq. At the time of the second blood draw, 3 of the 20 patients had radiological evidence of disease, while 17 patients did not. Of the three cases that did have radiological evidence of disease, one or more mutations was identified in the plasma of each patient. With the caveat of only a small number of patients presenting with recurrent disease, these findings suggest that liquid biopsy by ddPCR or RACE-Seq has a positive predictive value of 100% and a false negative rate of 0% during longitudinal follow-up of our patient cohort.

**Figure 4.**
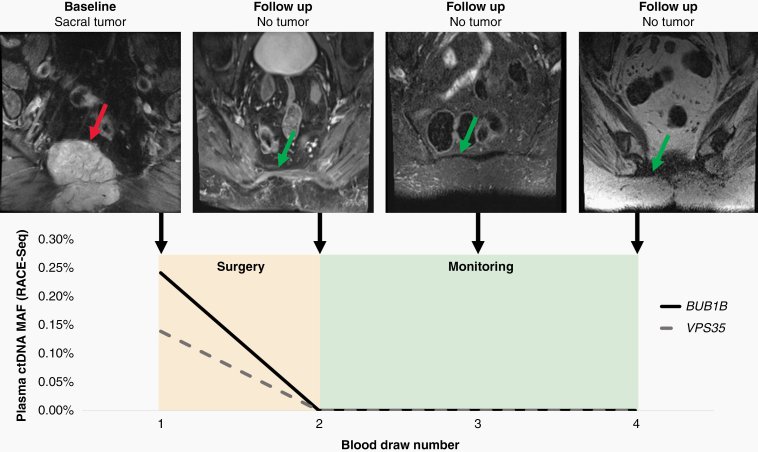
Liquid biopsy using RACE-Seq to detect mutations in *BUB1B* and *VPS35* demonstrates concordance with radiographic monitoring during the follow-up period for patient 8564. At the time of diagnosis, RACE-Seq detected mutations in *BUB1B* and *VPS35* that were concordant with whole exome sequencing data from the primary sacral tumor. After surgical resection, patient 8564 returned for three additional follow-up visits where no mutations were detected in plasma, which correlates with no detectable recurrence by MRI. The red arrow identifies the primary sacral tumor, while the green arrows demonstrates no tumor recurrence at the primary site.

**Figure 5.**
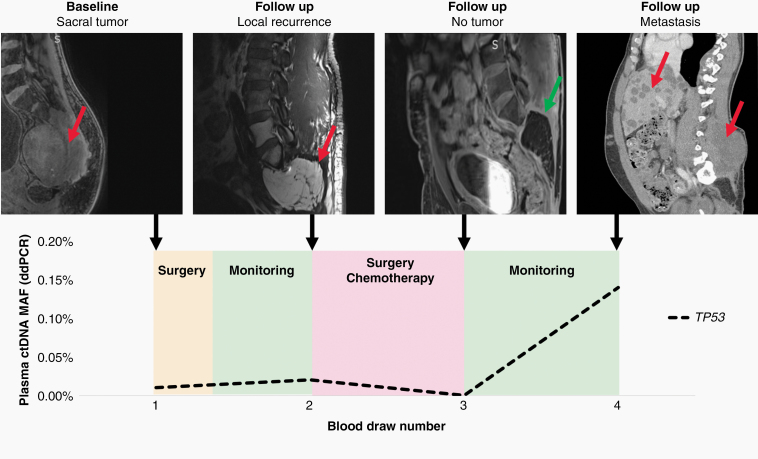
Liquid biopsy using ddPCR to detect mutations in *TP53* demonstrates concordance with radiographic monitoring during the follow-up period for patient 8134. At the time of diagnosis, ddPCR detected a mutation in *TP53* that was concordant with the whole exome sequencing data from the primary sacral tumor. Despite surgery, the patient presented with a locally recurrent tumor at the time of the second blood draw, which was reflected by a positive liquid biopsy result. After a second surgery, stereotactic radiotherapy, and chemotheray with imatinib and nivolumab, the patient's MRI was negative. When the patient returned for a final visit after being lost to follow-up for over a year, the patient had developed local recurrence and metasatic disease to the liver and lungs, which was detected by ddPCR. The red arrows identify tumor, while the green arrow shows no tumor recurrence at the primary site.

Patient 8564 had blood draws at the time of surgery and during three follow-up visits at Johns Hopkins. At each visit, plasma was collected and amplified by RACE-Seq for mutations in *BUB1B* and *VPS35* (Figure 4). RACE-Seq detected the two mutations in plasma at the time of surgery, which correlated with presence of the primary tumor by MRI. During the next three visits, RACE-Seq did not detect either mutation, which correlated with MRI scans demonstrating no radiographic evidence of recurrence. ddPCR was used to detect a TP53 mutation in patient 8134 at the time of surgery and later had radiographic local recurrence noted at the time of the second blood draw, which corresponded to a positive ctDNA result (Figure 5). He subsequently began systemic therapy with imatinib, followed by nivolumab as part of a clinical trial. At the time of the third blood draw after multiple rounds of system therapy, his ctDNA level was undetectable and MRI showed post-surgical changes but no residual tumor. He returned for follow-up nearly a year later and was found to have metastatic disease to the liver and lungs, in addition to recurrence at the primary site, and a positive ctDNA level. While our longitudinal analysis is limited by the number of samples collected at follow-up visits, these results suggest liquid biopsy may be useful for monitoring for disease recurrence of spinal chordomas.

Because RACE-Seq allows for the detection of multiple mutations at each follow-up visit, we were interested in whether the mutation with the greatest MAF in the tumor by WES was also the greatest in ctDNA at each time point. In five patients where we had longitudinal data beyond the initial blood draw that was analyzed by RACE-Seq, the same mutation that was at the greatest MAF in WES was also the greatest MAF at all time points for two of the five patients. For the other three patients, the mutation with the greatest MAF in WES was not the greatest MAF in subsequent blood draws but was nonetheless still identified. This finding highlights the possibility that, in parallel with potential recurrence and/or clonal evolution of the tumor, the fractions of various mutations in the ctDNA fluctuate over time. Thus, it may be important to utilize a detection technology such as RACE-Seq that captures the MAFs of multiple mutations over time.

Seventeen of the twenty patients who had two or more blood draws presented without evidence of disease. In 13 of these 17 cases, the patient-specific mutation was not detected in ctDNA, giving a liquid biopsy approach a specificity of 76% (Supplementary Table 1). Aside from the 3 cases with proven recurrence, 4 cases were also positive by liquid biopsy but had no radiographic evidence of disease, which suggests a false positive rate of 24%.

Of the 4 cases that were presumably false positives at the time of the second blood draw, patient 8468 had additional blood draws beyond the second. Each of the third and fourth blood draws in this patient had detectable levels of ctDNA mutations with no corresponding evidence of recurrence by MRI or CT scans. Including these two samples, there were seven longitudinal plasma samples in which a ctDNA was positive via RACE-Seq; however, none of these patients had clinical evidence of disease recurrence. To further rule out disease in these patients and confirm that detection of ctDNA mutations constituted false positives, we utilized an orthogonal approach called RealSeqS that detects levels of aneuploidy, a hallmark of nearly all cancers. None of the seven cases were found to have ctDNA demonstrating aneuploidy.

While the sensitivity of RACE-Seq has been reported to be higher than the sensitivity of RealSeqS, it is unclear why those plasma samples had detectable levels of ctDNA. One possibility could be that the RACE-Seq results are false positives, though nearly all samples had more than one alteration that was detectable. The concordance across mutations also makes false positives less likely. Alternatively, these mutations could be from background genomic DNA contamination and alterations found in hematopoietic cells, also known as clonal hematopoiesis of indeterminant potential (CHIP). This too seems less likely as RealSeqS has the capacity to identify lymphocytic DNA contamination and none of the cases were found to have elevated levels of genomic DNA. Another possibility is that these patients have microscopic recurrence that is not yet clinically detectable. Other groups have demonstrated that ctDNA can be used to determine minimal residual disease and recurrence prior to conventional imaging.^17,18^

Liquid biopsy also has the potential for false negative results. For example, patient 8381 had a pre-surgery ddPCR result that was negative, despite a 36 mm^3^ locally recurrent tumor. Similarly, patient 8455 did not have detectable levels of ctDNA at the time of surgery, despite a 154 mm^3^ primary tumor. One possibility for these false negatives is that we only assayed for one mutation in the plasma by ddPCR based on the MAF identified by WES and biological consequence of the mutation. It is possible that the other mutations identified in the primary tumor would be detected by a multiplex assay, such as RACE-Seq. Another explanation for the false negative results could be abnormally high background at the genomic location of the mutation of interest, which would limit the signal-to-noise ratio and decrease confidence in calling a mutation positive. While neither mutation in patient 8381 nor patient 8455 is found in traditional sequence contexts that are prone to increased PCR amplification and/or next generation sequencing errors, such as homopolymer repeats or regions of abnormally high GC content,^19^ we cannot eliminate the possibility that high background could limit sensitivity analysis. These possibilities further underscore the importance of assaying for multiple mutations at initial diagnosis and follow-up.

The median progression free survival for spinal chordoma is 6 years, and given the mean follow-up of 24 months, we cannot definitively identify all cases that may have recurred.^3^ Additionally, because only one death had occurred at the time of last follow-up, it was not possible to compare overall survival between ctDNA positive and ctDNA negative patients.

## Discussion

Our results demonstrate that 87.1% of tumors have detectable levels of ctDNA in patients with spinal chordomas, with MAFs ranging from less than 1% to over 29%. While access to longitudinal blood draws was limited in our cohort by the decision of many patients to seek follow-up care closer to home, we estimate that liquid biopsies may aid in the surveillance of spinal chordomas for recurrence. At the time of the second blood draw, we detected 100% of recurrent cases, with a specificity of 75% and a false positive rate of 25%.

In addition to demonstrating that ctDNA is a possible diagnostic tool for patients with chordoma, we reaffirm the findings of recurrent alterations in the epigenetic machinery.^16,20^ As presented in Figure 2, patients whose plasma was positive for mutations in ctDNA that were concordant with their primary tumor had greater MAFs in the primary tumor compared to those that were negative for mutations in ctDNA (*P* = .004). Given the multiplex nature of our assays, we included all mutations identified in a patient's primary tumor in Figure 2 to allow for full visualization of the distribution of MAFs detected in the primary tumors of ctDNA positive and negative patients.

Twenty-five percent of tumors tested had a mutation in genes that regulate epigenetic status across the genome, including mutations in genes responsible for chromatin remodeling and histone methylation. Most notable were mutations in *ARID1A, ARID1B,* and *PBRM1*, key members of the SWI/SNF and PBAF complexes that play a role in chromatin structure and transcriptional regulation.^21^ Functions of the SWI/SNF complex include nucleosome mobilization at promoters and enhancers, facilitating the binding of transcription factors, recruiting coactivators and corepressors, recruiting histone modifying enzymes, and facilitating chromatin looping to increase interaction between enhancers and promoters. *ARID1A* and other members of the SWI/SNF complex have been shown to have tumor suppressor functions, as loss of *ARID1A* leads to increased cellular proliferation, de-differentiation, and inhibition of apoptosis.^22^ Loss of *ARID1A* or other SWI/SNF members may also lead to transcriptional dysfunction, including disruption of nucleosome sliding activity, affecting the ability of the RNA polymerase II complex to bind promoters.

In addition, we described the mutational landscape of spinal chordomas in one of the largest cohorts published to date, strengthening the conclusions from the Tarpey et al. study^16^ that included 26 spinal chordomas in their whole genome or exome sequencing cohort, and highlighting mutations in tumor suppressor genes and genes that prevent aneuploidy at cell division. We found that the average mutation rate was 0.05/megabase, with a median of 5.5 mutations per sample. The most common type of mutation was a single base pair substitution (92%), which most frequently resulted in a non-synonymous coding change (79%). Pathway analysis demonstrated that the most commonly dysregulated pathway was chromatin remodeling.

One of the major clinical challenges in caring for individuals with chordoma is to be able to predict recurrence or resistance. Currently, only anatomic and PET imaging has been shown to be effective. Use of PET imaging to track recurrence additionally requires that the primary chordoma is PET positive before treatment and necessitates the use of radionuclides. Unfortunately, both are often obscured by extensive post-operative and post-treatment changes that occur after radical surgical procedures, spinal instrumentation and intensive radiation regimens. Therefore, we examined whether somatic mutations could be harnessed to develop personalized biomarkers for these patients. Of the 32 patients studied, all had putative somatic mutations which were queried in the matched plasma.

One of the main limitations of our study is the lack of follow-up blood draws at fixed intervals. While we are able to make some inferences about ctDNA levels and disease progression and response to treatment, it will be important for future prospective studies to collect additional blood samples over a longer period of time to determine whether ctDNA positivity predicts recurrence and overall survival. However, these initial results suggest that ctDNA holds promise as a personalized biomarker for chordomas, which are challenging to treat and have high rates of recurrence.

## Supplementary Material

vdaa173_suppl_Supplementary_Figure_1

vdaa173_suppl_Supplementary_Tables

vdaa173_suppl_Supplementary_Materials_1

vdaa173_suppl_Supplementary_Legends

## Notes

**Authorship Statement.** Experimental Design: A.K.M., B.Y., C.D., C.B., Y.J., C.B. Analysis and Interpretation of Data: A.K.M., B.Y., C.D., S.-fu L., D.S., J.P.W., Z.L.G., J.R., C.B., Y.J., C.B. Writing Manuscript: A.K.M., B.Y., C.D., S.-fu L., D.S., J.P.W., Z.L.G., J.R., C.B., Y.J., C.B.
