# Supplementary figures and images for: The mutational landscape of spinal chordomas and their sensitive detection using circulating tumor DNA
Source: Neurooncol Adv. 2020 Dec 8;3(1):vdaa173. doi: 10.1093/noajnl/vdaa173 (PMC7850091; doi:10.1093/noajnl/vdaa173)

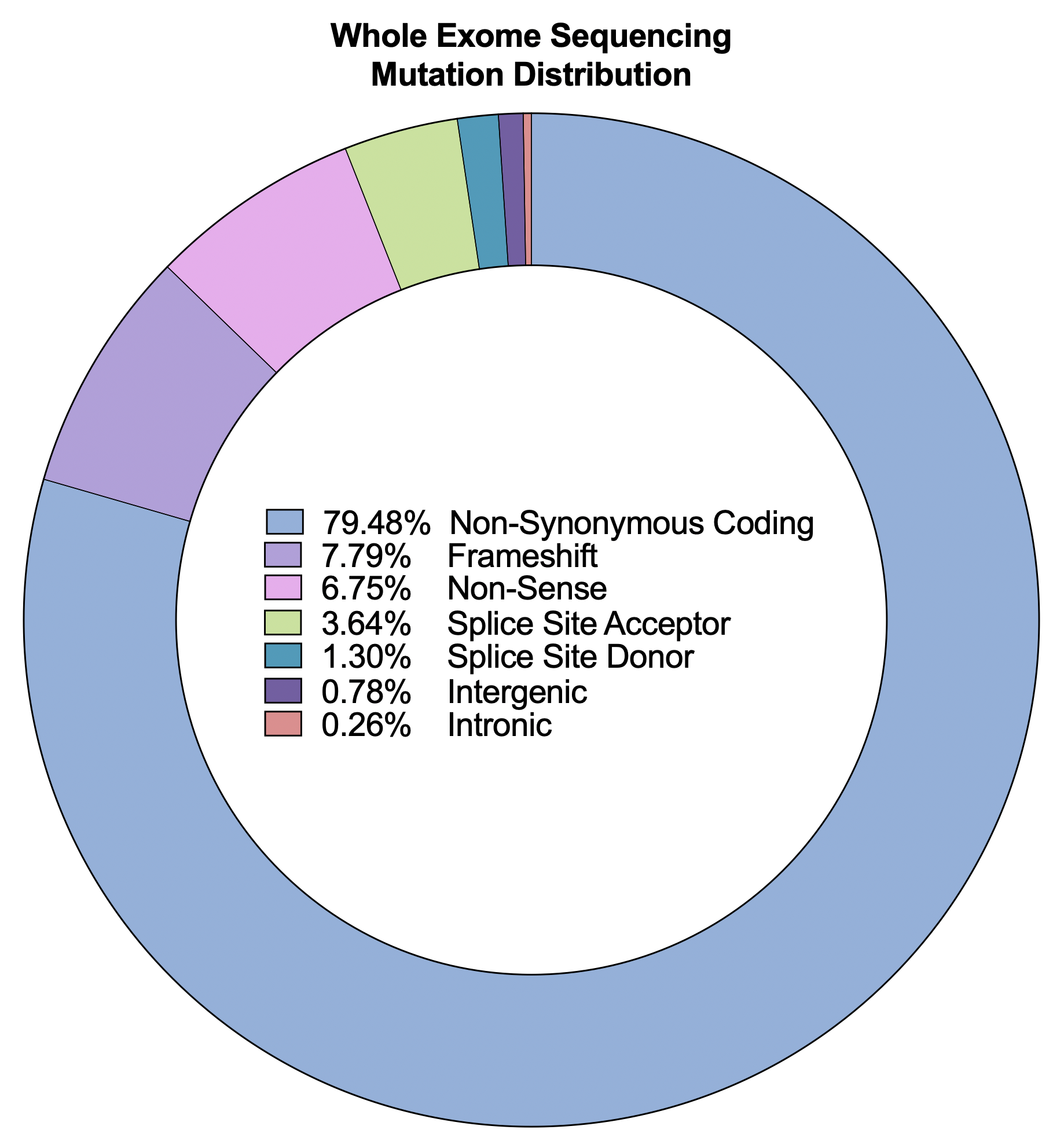

Supplement: vdaa173_suppl_Supplementary_Figure_1 [file vdaa173_suppl_supplementary_figure_1.png]
