# Supplementary material for: The mutational landscape of spinal chordomas and their sensitive detection using circulating tumor DNA
Source: Neurooncol Adv. 2020 Dec 8;3(1):vdaa173. doi: 10.1093/noajnl/vdaa173 (PMC7850091; doi:10.1093/noajnl/vdaa173)
Supplement: vdaa173_suppl_Supplementary_Materials_1 [file vdaa173_suppl_supplementary_materials_1.docx]

*Library Construction and Whole Exome Sequencing* *Buffer and PCR Conditions*

DNA was purified using Agencourt AMPure XP beads (Beckman Coulter, IN) in a ratio of 1.0 to 0.9 of PCR product to beads. Purified, fragmented DNA was mixed with 36 µl of H_2_O, 10 µl of End Repair Reaction Buffer, 5 µl of End Repair Enzyme Mix (cat# E6050, NEB, Ipswich, MA). The 100 µl end-repair mixture was incubated at 20°C for 30 min, and purified using Agencourt AMPure XP beads (Beckman Coulter, IN) in a ratio of 1.0 to 1.25 of PCR product to beads. 42 µl of end-repaired DNA was mixed with 5 µl of 10X dA Tailing Reaction Buffer and 3 µl of Klenow (exo-)(cat# E6053, NEB, Ipswich, MA). The 50 µl mixture was incubated at 37°C for 30 min and purified using Agencourt AMPure XP beads (Beckman Coulter, IN) in a ratio of 1.0 to 1.0 of PCR product to beads. 25 µl of A-tailed DNA was mixed with 6.7 µl of H_2_O, 3.3 µl of PE-adaptor (Illumina), 10 µl of 5X Ligation buffer and 5 µl of Quick T4 DNA ligase (cat# E6056, NEB, Ipswich, MA). The ligation mixture was incubated at 20°C for 15 min and purified using Agencourt AMPure XP beads (Beckman Coulter, IN) in a ratio of 1.0 to 0.95 and 1.0 of PCR product to beads.

To obtain an amplified library, twelve PCRs of 25 µl each were set up, each including 15.5 µl of H_2_O, 5 µl of 5 x Phusion HF buffer, 0.5 µl of a dNTP mix containing 10 mM of each dNTP, 1.25 µl of DMSO, 0.25 µl of Illumina PE primer #1, 0.25 µl of Illumina PE primer #2, 0.25 µl of Hotstart Phusion polymerase, and 2 µl of the DNA. The PCR program used was: 98°C for 2 minutes; 12 cycles of 98°C for 15 seconds, 65°C for 30 seconds, 72°C for 30 seconds; and 72°C for 5 min. DNA was purified using Agencourt AMPure XP beads (Beckman Coulter, IN) in a ratio of 1.0 to 1.0 of PCR product to beads. Exonic regions were captured in solution using the Agilent SureSelect v.4 kit (Agilent, Santa Clara, CA). The captured library was then purified with a Qiagen MinElute column purification kit and eluted in 17 µl of 70°C EB to obtain 15 µl of captured DNA library. To amplify the captured DNA library, eight 30 µL PCR reactions containing 19 µl of H_2_O, 6 µl of 5 x Phusion HF buffer, 0.6 µl of 10 mM dNTP, 1.5 µl of DMSO, 0.30 µl of Illumina PE primer #1, 0.30µl of Illumina PE primer #2, 0.30 µl of Hotstart Phusion polymerase, and 2 µl of captured exome library were set up. The PCR program used was: 98°C for 30 seconds; 14 cycles of 98°C for 10 seconds, 65°C for 30 seconds, 72°C for 30 seconds; and 72°C for 5 min. To purify PCR products, a NucleoSpin Extract II purification kit (Macherey-Nagel, PA) was used. Paired-end sequencing resulting in 100 bases from each end of the fragments was performed using Illumina HiSeq 2500 (Illumina, San Diego, CA).
