# Supplementary material for: The mutational landscape of spinal chordomas and their sensitive detection using circulating tumor DNA
Source: Neurooncol Adv. 2020 Dec 8;3(1):vdaa173. doi: 10.1093/noajnl/vdaa173 (PMC7850091; doi:10.1093/noajnl/vdaa173)
Supplement: vdaa173_suppl_Supplementary_Legends [file vdaa173_suppl_supplementary_legends.docx]

**Supplementary Figure 1.** The mutational landscape of primary spinal chordomas consists mostly of non-synonymous amino acid changes, in addition to frameshift and non-sense mutations that lead to protein truncation.

**Supplementary Table 1.** Detailed clinicopathological data for the study patient population

**Supplementary Table 2.** Sequences of the primers and probes used in ddPCR and RACE-Seq assays

**Supplementary Table 3.** Mutations identified by whole exome sequecning in the primary tumors of the study patients

**Supplementary Table 4.** Detailed results of the ddPCR and RACE-Seq assays for each patient
